# ﻿Updated species checklist of fishes from Lake Dongting in Hunan Province, South China: Species diversity and conservation

**DOI:** 10.3897/zookeys.1108.79960

**Published:** 2022-06-23

**Authors:** Xiao Chen, Man Wang, E Zhang

**Affiliations:** 1 The Key Laboratory of Aquatic Biodiversity and Conservation, Institute of Hydrobiology, Chinese Academy of Sciences, Wuhan, China Institute of Hydrobiology, Chinese Academy of Sciences Wuhan China; 2 University of Chinese Academy of Sciences, Beijing, China University of Chinese Academy of Sciences Beijing China

**Keywords:** Annotated list, biodiversity, ichthyofauna, taxonomy, threatened species

## Abstract

A lack of an updated checklist of freshwater fish species from Lake Dongting is a great hindrance to further biodiversity analysis. A seasonal survey of fishes in the lake was conducted from October 2017 to January 2019. Based on the data obtained during the field survey and coupled with known literature and the latest taxonomic development of relevant taxa, the species checklist of fishes from Lake Dongting was updated. A total of 130 species from 12 orders, 30 families and 76 genera has been documented, containing 126 native species and four alien species. Its fish fauna is dominated by the Xenocyprididae that has the highest number of included species (30), followed by the Gobionidae (25) and Acheilognathidae (11). This checklist comprises 20 species undergoing nomenclatural changes and 11 new records, eight of which are native and three exotic. It excludes 20 species, which have been reported in error in historical works, due to synonyms, erroneous records, taxonomic changes and unconfirmed records. Unsampled in this survey were 34 species that are ecologically specialised: migratory, rheophilic, predatory, shellfish-dependent or pelagic-egg-spawning. While some of these species eluded capture likely due to the paucity of population, others may have been extirpated in Lake Dongting perhaps owing to human perturbations, such as river damming across affluents or the Chang-Jiang mainstem, sand dredging, overfishing or water pollution. The updated checklist lays a sound foundation for biodiversity conservation of fishes in Lake Dongting.

## ﻿Introduction

Freshwater ecosystem and freshwater fish may well face one of the greatest threats in the world in the context of global biodiversity crisis (Dudgeon et al. 2006; Strayer and Dudgeon 2010). In comparison with other vertebrates, freshwater fishes are being more severely threatened by human interferences when the usage of water resources is strengthened (Reid et al. 2019; Barbarossa et al. 2021). The biodiversity conservation of freshwater fishes, nevertheless, has received disproportionate attention compared to terrestrial vertebrates (Tedesco et al. 2017). It is imperative and also in urgent need to protect the freshwater ecosystem and its biodiversity (Jackson et al. 2001; Liermann et al. 2012). Species inventories are beyond simply lists of names; they are actually representing an efficient method for obtaining updated information regarding species composition and distributions (Marta et al. 2019). This information provides valuable inputs of biodiversity monitoring which serves many conservational purposes, such as prioritising protection areas and directing conservation actions (Brooks et al. 2004). An update species inventory of a given area is of vital significance for biodiversity conservation.

The freshwater ecosystem of the Chang-Jiang (= Yangtze River; Jiang, Shui and He in Chinese mean river), the third largest river of the world and the largest river of China, supports rich biodiversity of aquatic organisms (Chen et al. 2020). The middle reaches of this river are regarded as one of the hotspots for freshwater fish diversity in Asia (Kottelat and Whitten 1996). Lake Dongting is one of two largest river-linked freshwater lakes in China, lying within the lower Chang-Jiang (= the mid-lower Chang-Jiang) basin which forms a freshwater ecoregion of the world for biodiversity conservation (Abell et al. 2008). This lake is an important portion of fluvio-lacustrine complex ecosystems of the mid-lower Chang-Jiang basin (Wang et al. 2019b), and also one of the priority areas for biodiversity conservation in China (Li et al. 2016a) and the Ramsar-listed floodplain wetlands (Dong et al. 2021), which serves as crucial habitats of migratory birds of East Asia-Australian flyway (Zou et al. 2019). Moreover, Lake Dongting, as a flood buffer zone, provides *Elaphurusdavidianus* (Milu or Père David’s deer) with seasonal sanctuaries (Yang et al. 2016). It also provides refuge for charismatic mammals like *Neophocaenaasiaeorientalis* (Yangtze finless porpoise) (Zhang 2011; Huang et al. 2017) and the feeding grounds of large-sized flagship fishes, such as *Acipensersinensis* (Chinses sturgeon) and *Psephurusgladius* (Chinese paddlefish) and economically-important potamodromous fishes like four major Chinese carps: *Aristichthysnobilis* (Bighead Carp), *Ctenopharyngodonidella* (Grass Carp), *Hypophthalmichthysmolitrix* (Silver Carp) and *Mylopharyngodonpiceus* (Black Carp) (Liu et al. 2010; Zhang et al. 2020a). Apparently, the lake plays a vital role in the conservation of aquatic biodiversity of the Chang-Jiang Basin.

The aquatic biodiversity of Lake Dongting is greatly imperilled by anthropogenic activities, like sand dredging, overfishing, alien species invasion, water pollution from industrial, agricultural and domestic sewage discharges and so forth (Dou and Jiang 2000; Fu et al. 2021; Jiang et al. 2022). It is also indirectly impacted by dam building across the Chang-Jiang mainstem and affluents of the lake owing to the continuity of the aquatic ecosystem (Wang et al. 2016; Liu and Wang 2018). Fishes, as top feeders of the aquatic ecosystem and an important source of proteins in human food, are severely threatened by these factors (Zhao et al. 2019; Tregidgo et al. 2021). In the latest Red List assessment of Chinese freshwater fishes (Zhang and Cao 2021a), there are 12 imperilled species from Lake Dongting. *Psephurusgladius* (Martens, 1862) was recently declared to be extinct or functionally extinct (Zhang et al. 2020a). Such species as *Luciobramamacrocephalus* (Lacepède, 1803), *Ochetobiuselongatus* (Kner, 1867) and *Tenualosareevesii* (Richardson, 1846) have been not seen in capture fisheries for decades or are likely extirpated (Wu et al. 2015; Zhang and Cao 2021b). The current status of freshwater fish diversity of Lake Dongting is, therefore, of particular conservation concern.

An updated checklist of fishes from Lake Dongting remains to be provided. The first checklist of freshwater fishes from this lake was given by Tang and Qian (1979), who recorded 114 fish species. A total of 117 species of the lake was later included in Anonymous’ (1980) book entitled “Fish of Hunan Province”. Subsequent species inventories of fishes from Lake Dongting primarily followed the book and three authoritative monographs of Chinese freshwater fishes authored by Chen (1998), Chu et al. (1999) and Yue (2000). Nevertheless, the species inventory of fishes from this lake needs to be regularly updated for biodiversity conservation, especially with lots of taxonomic revisions of freshwater fishes from the Chang-Jiang basin over the past decades. Therefore, three seasonal field sampling of fishes in Lake Dongting were conducted by us during 2017–2019. Coupled with the data collected in this survey, we aim to synthesise existing knowledge of freshwater fish diversity and systematics to provide an updated checklist of fish of the lake.

## ﻿History of taxonomic research

The taxonomic history of fishes from Lake Dongting could be traced back to the mid-19^th^ century. Père Heude, a French Jesuit catholic priest, made a collection of fish specimens at the lake from 1869 to 1884 (Luo 2005). Subsequently, Kreyenberg and Pappenheim (1908) reported 22 species from the Chang-Jiang and its tributaries, two of which were new species from Lake Dongting: *Coiliabrachygnathus* Kreyenberg & Pappenheim, 1908 and *Culteroxycephaloides* Kreyenberg & Pappenheim, 1908. At the same time, Regan (1908) described three new Chinese species, two of which were *Glyptothoraxsinensis* (Regan, 1908) and *Hemisalanxprognathus* Regan, 1908 from the lake. In 1921, Clifford Pope made a collection of fish specimens in Huping College, Yochow (= Yueyang City near East Lake Dongting) (Luo 2005). Nichols (1925a) proposed two new subspecies *Misgurnusanguillicaudatustungting* Nichols, 1925 and *Misgurnusmohoityleopardus* Nichols, 1925 from Lake Dongting, both being regarded as invalid to date. Nichols (1925b) recorded three species of *Botia* Gray, 1831 from this lake, two of which were new to sciences, namely *B.citrauratea* Nichols, 1925 and *B.purpurea* Nichols, 1925. Both are now placed in *Leptobotia* Bleeker, 1870, but the latter has been synonymised with *L.taeniops* (Sauvage, 1878). Simultaneously, Nichols (1925c-e) described five new species from Lake Dongting, viz. *Gobiolongipinnis* (= *Rhinogobioventralis* Sauvage & Dabry de Thiersant, 1874), *Gobiuscliffordpopei* [= *Rhinogobiuscliffordpopei* (Nichols, 1925)], *Hemiculterellaengraulis* [= *Pseudolaubucaengraulis* (Nichols, 1925)], *Hemiculturclupeoides* [= *Hemiculterleucisculus* (Basilewsky, 1855)] and *Varicorhnustungting* [= *Decorustungting* (Nichols, 1925)]. Later, Nichols and his co-authors (1926, 1927) named a new subspecies *Sarcocheilichthysnigripinnistungting* Nichols & Pope, 1927 and two new species, i.e. *Acheilognathusgracilis* Nichols, 1926 and *Pseudogobiotungtingensis* [= *Microphysogobiotungtingensis* (Nichols, 1926)] from this lake. Nichols (1928) recorded 71 nominal species from Lake Dongting in his provisional checklist of Chinese freshwater fishes. Subsequent taxonomic contributions to fishes of Lake Dongting were also made by many authors, such as Wu (1930), Tchang (1933) and Kimura (1934), who made a small collection of fish specimens in the lake. Chu (1931) recorded 74 fish species from Hunan Province, the majority of which were from Lake Dongting. Hora (1932) described a new species *Lepturichthysnicholsi* [= *Lepturichthysfimbriatus* (Günther, 1888)]. Nichols (1943), in his book entitled “The fresh-water fishes of China”, recognised 79 species or subspecies for fish specimens collected by Clifford Pope in East Dongting Lake in 1921.

More studies were focused on the species inventory of fishes from Lake Dongting following the establishment of P. R. China in 1949. Forty-three species of the lake were involved in Chu’s (1955) study on the distribution of fish species in the Yichang section of the Chang-Jiang. Liang and Liu (1959) compiled a species list of 69 fishes from Lake Dongting and its affluent (Xiang-Jiang). Later, Liang and Liu (1966), in their checklist of fishes from Hunan Province, recorded 119 species from Lake Dongting. Anonymous (1976) also reported 84 species from Lake Dongting in the book entitled “Fishes of the Chang-Jiang”. Tang and Qian (1979) were the first to provide a checklist of 114 fish species or subspecies from the lake. Anonymous (1980) included 117 species from Lake Dongting in the book entitled “Fishes of Hunan Province”. Although Dou and Jiang (2000) compiled a checklist of 104 species from the lake, this work was mainly based on their collections of fish specimens made during 1974–1975.

As from the 1990s, increasing research interests have centred on the fish diversity of Lake Dongting. Survey of fishery resources carried out by Liao et al. (2002), Liao et al. (2006) and Li (2006) into Lake Dongting from 1994 to 2005 found 117, 111 and 117 species, respectively. Ru and Liu (2013) identified 69 species in their surveys conducted into East and South Dongting Lake during 2004–2005. Li (2013) reported a total of 85 fish species, based on his field sampling from March to December in 2012. Eighty species were identified by Jiang et al. (2019) in their research on the spatio-temporal patterns of fish assemblages in Lake Dongting from 2012 to 2014. Sixty-two fish species were recorded by Qin et al. (2019) from the outlet channel of the lake from 2013 to 2015. Eighty-five and 66 fish species were sampled during 2002–2003 and 2012–2014 field surveys to monitor the changes of fish community structure at West Dongting Lake before and after the operation of Three Gorges Dam (Zhu et al. 2014). All these inventories were conducted, particularly in relation to environment impact assessment prepared for hydropower projects or fisheries investigations and the data collection used for biodiversity analyses. Some surveys were focused on fish resources assessment; small-sized or less commercially valuable species were largely neglected. Others were not based on examination of collected specimens, but compiled through desk review or interview, containing little or no reliable information on fish diversity, although they claimed to have studied biodiversity. More importantly, these inventories required critical scrutiny from an ichthyological perspective as most of them, if not all, were not conducted by trained ichthyologists in the field; information on unrecognised species is impossibly captured through these surveys, therefore giving rise to a grossly underestimated biodiversity value and taxonomic impediment (Sluys 2013).

## ﻿Material and method

Lake Dongting (28°44'N–29°35'N, 111°53'E–113°05'E) is located in the northern part of Hunan Province, connected to the middle Chang-Jiang mainstem (Wang and Dou 1998). This water-carrying floodplain lake receives not only runoff waters from four main affluents (Xiang-Jiang, Zi-Shui, Yuan-Jiang and Li-Shui), but flood water from the Chang-Jiang mainstem via three inlet channels (Songzi, Hudu and Ouchi Channel) and lake water then flows out into the mainstem of the river again via Chenglingji Channel (Dou and Jiang 2000). Generally, Lake Dongting is divided into three sub-lakes, i.e. East Dongting Lake, South Dongting Lake and West Dongting Lake (Zhao et al. 2005). The lake covers a surface area of 2625 km^2^ at a water level of 33.5 m at Chenglingji Station (Dou and Jiang 2000).

**Figure 1.**
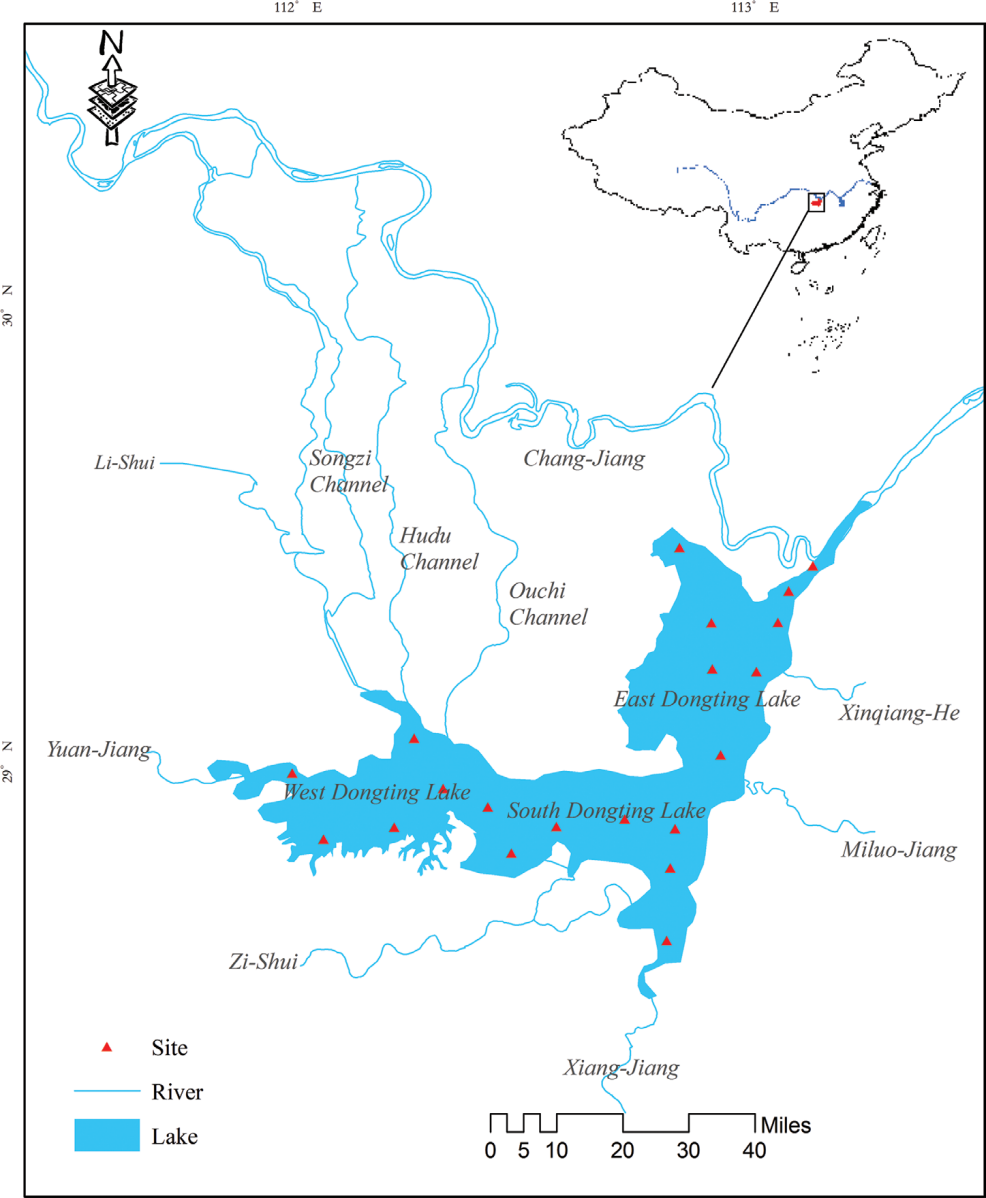
Field sampling sites of Lake Dongting in this study.

Twenty sampling sites were selected in this study, based on habitat heterogeneity (Fig. 1; Suppl. material 1: Table S1). Field sampling was taken from October to November 2017, July to August 2018 and December 2018 to January 2019. Fish surveys were conducted in different types of habitats to ensure maximum representation of species diversity occurring in this area. Multiple sampling methods were thus applied. Three-layer gill nets were used for pelagic fish sampling, while trap nets were applied to catch demersal fishes. Additionally, fish specimens were collected from local fish markets and their sampling localities were restricted to Lake Dongting.

All collected specimens were identified to species level. The initial identification in the field principally followed Chen (1998), Chu et al. (1999) and Yue (2000). The caught specimens were fixed in 95% alcohol solution for molecular analysis, in general and DNA extraction, in particular or initially fixed in 10% formalin and then transferred to 70% ethanol for morphological examination and also for permanent collection. All specimens were deposited in the ichthyolgical collection of the Museum of Aquatic Organisms at the Institute of Hydrobiology, Chinese Academy of Sciences.

In addition to the data collected during our field sampling, known research works were referred. Reference was made to the following main historical records of fishes in the lake: Liang and Liu (1959, 1966), Tang and Qian (1979), Anonymous (1980), Li (2006), Ru (2008), Cao et al. (2012) and Li (2013).

Fish classifications are being transformed greatly as latest molecular phylogenies provide evidence in support for natural groups which were unanticipated by previous studies (Betancur-R et al. 2017). For the order Cypriniformes, the dominant group of freshwater fishes worldwide, significant advances have been made in its familial-level classification; some new familial (Acheilognathidae, Gobionidae and Xenocyprididae) and subfamilial names (Acrossocheilinae and Spinibarbinae) have been proposed (Tan and Armbruster 2018). Except for Cypriniformes, the taxonomic revisions of other orders were referred from Van Der Laan et al. (2014). The species checklist of fishes in Lake Dongting was systematically arranged by order, family and subfamily in accordance with the latest developments made in the taxonomic ranks (Van Der Laan et al. 2014).

## ﻿Results

### ﻿Analysis of the species checklist

A total of 130 fish species, identified from 12 orders, 30 families and 76 genera, have been documented from Lake Dongting (Table 1). Amongst these species, there are 126 native and four exotic species. Ninety-six species from 10 orders, 24 families and 61 genera collected during 2017–2019 fish survey are included.

**Table 1.** Annotated checklist of the fish fauna from Lake Dongting. The species under each family or subfamily are sorted by alphabetical order. Notes are labelled with taxonomic alteration, synonymisation, misidentification and other meanings.

Valid species name	Previous studies	Note
**Acipenseriformes**		
**Acipenseridae**		
001 *Acipenserdabryanus* Duméril, 1869		○P
002 *Acipensersinensis* Gray, 1835		○D
**Polyodontidae**		
003 *Psephurusgladius* (Martens, 1862)		○D
**Anguilliformes**		
**Anguillidae**		
004 *Anguillajaponica* Temminck & Schlegel, 1846		⊕D
**Clupeiformes**		
**Clupeidae**		
005 *Tenualosareevesii* (Richardson, 1846)		○D
**Engraulidae**		
006 *Coiliabrachygnathus* Kreyenberg & Pappenheim, 1908		⊕P
007 *Coilianasus* Temminck & Schlegel, 1846		○D
**Cypriniformes**		
**Catostomidae**		
008 *Myxocyprinusasiaticus* (Bleeker, 1864)		⊕P
**Botiidae**		
009 *Leptobotiacitrauratea* (Nichols, 1925)		⊕P
	*Leptobotiaelongata* (Bleeker, 1870)	⊙M
010 *Leptobotiarubrilabris* (Dabry de Thiersant, 1872)		○P
011 *Leptobotiataeniops* (Sauvage, 1878)		⊕P
012 *Parabotiabanarescui* (Nalbant, 1965)		⊕P
013 *Parabotiafasciata* Dabry de Thiersant, 1872		⊕P
**Cobitidae**		
014 *Cobitismacrostigma* Dabry de Thiersant, 1872		⊕P
015 *Cobitissinensis* Sauvage & Dabry de Thiersant, 1874		⊕P
016 *Misgurnusanguillicaudatus* (Cantor, 1842)		⊕P
017 *Paramisgurnusdabryanus* Dabry de Thiersant, 1872		⊕P
**Balitoridae**		
018 *Lepturichthysfimbriatus* (Günther, 1888)		○P
**Cyprinidae**		
**Labeoninae**		
019 *Cirrhinuscirrhosus* Bloch, 1795		+AP
020 *Cirrhinusmolitorella* (Valenciennes, 1844)		⊕AP
021 *Decorustungting* (Nichols, 1925)	*Banganatungting* (Nichols, 1925)	○TP
022 *Pseudogyrinocheilusprochilus* (Sauvage & Dabry de Thiersant, 1874)		+P
**Cyprininae**		
023 *Carassiusauratus* (Linnaeus, 1758)		⊕P
	*Cyprinuscarpio* Linnaeus, 1758	⊙T
024 *Cyprinusrubrofuscus* Lacepède, 1803		⊕P
	*Procyprisrabaudi* (Tchang, 1930)	⊙M
**Acrossocheilinae**		
025 *Onychostomararum* (Lin, 1933)		○P
026 *Onychostomasimum* (Sauvage & Dabry de Thiersant, 1874)		○P
**Spinibarbinae**		
027 *Spinibarbuscaldwelli* (Nichols, 1925)		○P
	*Spinibarbushollandi* Oshima, 1919	⊙T
	*Spinibarbussinensis* Bleeker, 1871	⊙M
**Xenocyprididae**		
028 *Aristichthysnobilis* (Richardson, 1845)		⊕P
029 *Chanodichthysdabryi* (Bleeker, 1871)	*Culterdabryi* Bleeker, 1871	⊕TP
030 *Chanodichthyserythropterus* (Basilewsky, 1855)	*Culteralburnus* Basilewsky, 1855	⊕TP
031 *Chanodichthysmongolicus* (Basilewsky, 1855)	*Cultermongolicus* Basilewsky, 1855	⊕TP
032 *Chanodichthysoxycephalus* (Bleeker, 1871)	*Culteroxycephalus* Bleeker, 1871	○TP
033 *Chanodichthysoxycephaloides* (Kreyenberg & Pappenheim, 1908)	*Culteroxycephaloides* Kreyenberg & Pappenheim, 1908	⊕TP
034 *Culteralburnus* (Basilewsky, 1855)	*Cultrichthyserythropterus* (Basilewsky, 1855)	⊕TP
035 *Ctenopharyngodonidella* (Valenciennes, 1844)		⊕P
036 *Distoechodontumirostris* Peters, 1881		○P
037 *Elopichthysbambusa* (Richardson, 1845)		⊕P
038 *Hemiculterbleekeri* Warpachowski, 1888		⊕P
039 *Hemiculterleucisculus* (Basilewsky, 1855)		⊕P
040 *Hypophthalmichthysmolitrix* (Valenciennes, 1844)		⊕P
041 *Luciobramamacrocephalus* (Lacepède, 1803)		○P
042 *Megalobramaamblycephala* Yih, 1955		⊕P
043 *Megalobramamantschuricus* (Basilewsky, 1855)	*Megalobramaskolkovii* Dybowski, 1872	⊕SP
044 *Mylopharyngodonpiceus* (Richardson, 1846)		⊕P
045 *Ochetobiuselongatus* (Kner, 1867)		○P
046 *Opsariichthysbidens* Günther, 1873		⊕P
047 *Parabramispekinensis* (Basilewsky, 1855)		⊕P
048 *Plagiognathopsmicrolepis* (Bleeker, 1871)	*Xenocyprismicrolepis* Bleeker, 1871	○TP
049 *Pseudobramasimoni* (Bleeker, 1864)		⊕P
050 *Pseudolaubucaengraulis* (Nichols, 1925)		○P
051 *Pseudolaubucasinensis* Bleeker, 1864		⊕P
052 *Sinibramamacrops* (Günther, 1868)		⊕P
	*Sinibramawui* (Rendahl, 1933)	⊙S
053 *Squaliobarbuscurriculus* (Richardson, 1846)		⊕P
054 *Toxabramisswinhonis* Günther, 1873		⊕P
055 *Xenocyprisdavidi* Bleeker, 1871		⊕P
056 *Xenocyprismacrolepis* Bleeker, 1871	*Xenocyprisargentea* Günther, 1868	⊕SP
057 *Zaccoacanthogenys* (Boulenger, 1901)		⊕P
	*Zaccoplatypus* (Temminck & Schlegel, 1846)	⊙T
**Acheilognathidae**		
058 *Acheilognathusbarbatulus* Günther, 1873		+P
059 *Acheilognathusbarbatus* Nichols, 1926		○P
060 *Acheilognathuschankaensis* (Dybowski, 1872)		○P
061 *Acheilognathusgracilis* Nichols, 1926		⊕P
	*Acheilognathusimberbis* Günther, 1868	⊙U
062 *Acheilognathushypselonotus* (Bleeker, 1871)		○P
063 *Acheilognathusmacromandibularis* Doi, Arai & Liu, 1999		+P
064 *Acheilognathusmacropterus* (Bleeker, 1871)		⊕P
065 *Acheilognathuspolylepis* (Wu, 1964)		⊕P
066 *Acheilognathustonkinensis* (Vaillant, 1892)		○P
	*Acheilognathustaenianalis* (Günther, 1873)	⊙S
067 *Rhodeusocellatus* (Kner, 1866)		⊕P
068 *Rhodeussinensis* Günther, 1868		⊕P
**Gobionidae**		
069 *Abbottinarivularis* (Basilewsky, 1855)		⊕P
070 *Coreiusheterodon* (Bleeker, 1864)		⊕P
	*Coreiusguichenoti* (Sauvage & Dabry de Thiersant, 1874)	⊙M
071 *Gobiobotiafilifer* (Garman, 1912)		⊕P
072 *Gobiobotiameridionalis* Chen & Cao, 1977	*Gobiobotialongibarbameridionalis* Chen & Cao, 1977	⊕TP
073 *Gobiobotianicholsi* Bănărescu & Nalbant, 1966		○P
074 *Gobiobotialii* Chen, Wang, Cao & Zhang, 2022		+P
	*Gobiobotiapappenheimi* Kreyenberg, 1911	⊙M
	*Xenophysogobioboulengeri* (Tchang, 1929)	⊙M
075 *Hemibarbuslabeo* (Pallas, 1776)		⊕P
076 *Hemibarbusmaculatus* Bleeker, 1871		⊕P
077 *Microphysogobiotungtingensis* (Nichols, 1926)		⊕P
078 *Paracanthobramaguichenoti* Bleeker, 1864		⊕P
079 *Pseudogobiovaillanti* (Sauvage, 1878)		+P
080 *Pseudorasboraparva* (Temminck & Schlegel, 1846)		⊕P
081 *Rhinogobiocylindricus* Günther, 1888		○P
082 *Rhinogobiotypus* Bleeker, 1871		⊕P
083 *Rhinogobioventralis* Sauvage & Dabry de Thiersant, 1874		○P
	*Sarcocheilichthyskiangsiensis* Nichols, 1930	⊙T
084 *Sarcocheilichthysnigripinnis* (Günther, 1873)		⊕P
085 *Sarcocheilichthystungtingensis* Nichols & Pope, 1927		○P
086 *Sarcocheilichthyssinensis* Bleeker, 1871		⊕P
087 *Saurogobiodabryi* Bleeker, 1871		⊕P
088 *Saurogobiodumerili* Bleeker, 1871		○P
089 *Saurogobiogymnocheilus* Lo, Yao & Chen, 1998		⊕P
090 *Saurogobiogracilicaudatus* Yao & Yang, 1977		+P
091 *Saurogobiolissilabris* Bănărescu & Nalbant, 1973		⊕P
092 *Saurogobioxiangjiangensis* Tang, 1980		+P
093 *Squalidusargentatus* (Sauvage & Dabry de Thiersant, 1874)		⊕P
	*Squalidusnitens* (Günther, 1873)	⊙U
**Siluriformes**		
**Bagridae**		
094 *Hemibagrusmacropterus* Bleeker, 1870	*Mystusmacropterus* (Bleeker, 1870)	⊕P
095 *Tachysuruscrassilabris* (Günther, 1864)	*Leiocassiscrassilabris* Günther, 1864	⊕TP
096 *Tachysurusdumerili* (Bleeker, 1864)	*Leiocassislongirostris* Günther, 1864	⊕TP
097 *Tachysuruseupogon* (Boulenger, 1892)	*Pelteobagruseupogon* (Boulenger, 1892)	⊕TP
098 *Tachysurusmica* (Gromov, 1970)	*Leiocassisargentivittatus* (Regan,1905)	+TP
099 *Tachysurusnitidus* (Sauvage & Dabry de Thiersant, 1874)	*Pelteobagrusnitidus* (Sauvage & Dabry de Thiersant, 1874)	⊕TP
100 *Tachysurussinensis* (Lacepède, 1803)	*Pelteobagrusfulvidraco* (Richardson, 1846)	⊕TP
	*Tachysurustenuis* (Günther, 1873)	⊙M
101 *Tachysurusussuriensis* (Dybowski, 1872)	*Pseudobagrusussuriensis* (Dybowski, 1872)	○TP
102 *Tachysurusvachellii* (Richardson, 1846)	*Pelteobagrusvachellii* (Richardson, 1846)	⊕TP
103 *Tachysuruszhangfei* Shao, Cheng & Zhang, 2021	*Pseudobagrusalbomarginatus* (Rendahl, 1928)	⊕TP
**Amblycipitidae**		
104 *Liobagrusaequilabris* Wright & Ng, 2008		⊕P
**Sisoridae**		
105 *Glyptothoraxsinensis* (Regan, 1908)		⊕P
**Siluridae**		
106 *Silurusasotus* Linnaeus, 1758		⊕P
107 *Silurusmeridionalis* Chen, 1977		⊕P
**Ictaluridae**		
108 *Ictaluruspunctatus* (Rafinesque, 1818)		+AP
**Osmeriformes**		
**Salangidae**		
109 *Hemisalanxprognathus* Regan, 1908		⊕F
	*Hemisalanxbrachyrostralis* (Fang, 1934)	⊙S
110 *Neosalanxbrevirostris* (Pellegrin, 1923)		○F
	*Neosalanxtaihuensis* Chen, 1956	⊙S
111 *Neosalanxjordani* Wakiya & Takahashi, 1937		○F
	*Neosalanxoligodontis* Chen, 1956	⊙S
112 *Protosalanxhyalocranius* (Abbott, 1901)		○F
**Gobiiformes**		
**Odontobutidae**		
113 *Micropercopscinctus* (Dabry de Thiersant, 1872)	*Micropercopsswinhonis* (Günther, 1873)	⊕SV
114 *Odontobutissinensis* Wu, Chen & Chong, 2002		⊕V
**Gobiidae**		
115 *Mugilogobiusmyxodermus* (Herre, 1935)		○V
	*Rhinogobiusbrunneus* (Temminck & Schlegel, 1845)	⊙U
116 *Rhinogobiuscliffordpopei* (Nichols, 1925)		○V
117 *Rhinogobiussimilis* Gill, 1859		⊕V
	*Rhinogobiusgiurinus* Gill, 1859	⊙S
**Synbranchiformes**		
**Mastacembelidae**		
118 *Sinobdellasinensis* (Bleeker, 1870)		⊕P
**Synbranchidae**		
119 *Monopterusalbus* (Zuiew, 1793)		⊕P
**Anabantiformes**		
**Osphronemidae**		
120 *Macropodusopercularis* (Linnaeus, 1758)		⊕P
**Channidae**		
121 *Channaargus* (Cantor, 1842)		⊕P
122 *Channaasiatica* (Linnaeus, 1758)		⊕P
**Beloniformes**		
**Hemiramphidae**		
123 *Hyporhamphusintermedius* (Cantor, 1842)		⊕V
**Adrianichthyidae**		
124 *Oryziaslatipes* (Temminck & Schlegel, 1846)		○F
**Tetraodontiformes**		
**Tetraodontidae**		
125 *Takifuguobscurus* (Abe, 1949)		○D
**Centrarchiformes**		
**Centrarchidae**		
126 *Micropterussalmoides* (Lacepède, 1802)		+AP
**Sinipercidae**		
127 *Sinipercachuatsi* (Basilewsky, 1855)		⊕P
128 *Sinipercaknerii* Garman, 1912		⊕P
129 *Sinipercaroulei* Wu, 1930	*Coreosinipercaroulei* (Wu, 1930)	⊕TP
130 *Sinipercascherzeri* Steindachner, 1892		⊕P

**Note**: ⊕ Historically recorded species caught in this study; ○ Historically recorded species uncollected in this study; ⊙ Historically recorded species excluded from the updated checklist; + Newly-recorded species; A-Alien or introduced species; S-Junior synonym species; M-Previously misidentified species; T-Taxonomically altered species; U-Unconfirmed species; P-Primary freshwater species; F-Secondary freshwater species; D-Diadromous species; V-Vicarious species.

For species richness, the order representing the greatest number of species were Cypriniformes (86 species, 66.15% of the total), followed by the Siluriformes (15, 11.54%), Centrarchiformes (5, 3.85%), Gobiiformes (5, 3.85%), Osmeriformes (4, 3.08%), Anabantiformes (3, 2.31%), Clupeiformes (3, 2.31%), Acipenseriformes (3, 2.31%), Synbranchiformes (2, 1.54%), Beloniformes (2, 1.54%), Tetrodontiformes (1, 0.77%) and Anguilliformes (1, 0.77%). The family Xenocyprididae has the highest number (30) of fish species, accounting for 23.08% of the total, followed by the Gobionidae and Acheilognathidae, with 25 and 11 species contributing to 19.23% and 8.46%, respectively. The subsequent families included the Bagridae (10, 7.69%), Cyprinidae (9, 6.92%) and so forth (Table 2).

**Table 2.** The taxonomic composition of fish species in Lake Dongting.

Order	Family	Genus	Species
Acipenseriformes	Acipenseridae	1	2
	Polyodontidae	1	1
Anguilliformes	Anguillidae	1	1
Clupeiformes	Clupeidae	1	1
	Engraulidae	1	2
Cypriniformes	Catostomidae	1	1
	Botiidae	2	5
	Cobitidae	3	4
	Balitoridae	1	1
	Cyprinidae	7	9
	Xenocyprididae	22	30
	Acheilognathidae	2	11
	Gobionidae	12	25
Siluriformes	Bagridae	2	10
	Amblycipitidae	1	1
	Sisoridae	1	1
	Siluridae	1	2
	Ictaluridae	1	1
Osmeriformes	Salangidae	3	4
Gobiiformes	Odontobutidae	2	2
	Gobiidae	1	3
Synbranchiformes	Mastacembelidae	1	1
	Sybranchidae	1	1
Anabantiformes	Osphronemidae	1	1
	Channidae	1	2
Beloniformes	Hemiramphidae	1	1
	Adrianichthyidae	1	1
Tetraodontiformes	Tetraodontidae	1	1
Centrarchiformes	Centrarchidae	1	1
	Sinipercidae	1	4
12	30	76	130

Lake Dongting harboured 27 migratory fishes, six of which are diadromous and 21 potamodromous, and 103 sedentary fishes, accounting for 20.77% and 79.23% of the total freshwater fishes, respectively. There are 113 (86.92% of the total species) primary freshwater fishes (species spending the whole life in freshwater; Kottelat et al. (2012)), five (3.85%) secondary freshwater fishes (species related to marine families, but living in fresh or sometimes brackish water), six (4.62%) diadromous species (species migrating between fresh and brackish water, but staying in freshwater for part of their life), six (4.62%) vicarious species (species of otherwise largely marine families, but spending their whole life in freshwater, for example, some gobies species) (See Table 1).

The updated checklist of fishes from Lake Dongting includes 49 species endemic to China, 22 endemic to the Chang-Jiang and nine endemic to the mid-lower Chang-Jiang, respectively. This survey yielded 35 Chinese endemics (accounting for 71.43% of the total Chinese endemics from Lake Dongting), 13 endemic species of the Chang-Jiang (59.09% of the total endemic species of the river from the lake) and six endemic species of the mid-lower Chang-Jiang (66.67% of the total endemic species of these reaches from the lake), respectively.

### ﻿Annotated species checklist

The updated checklist of fishes in Lake Dongting recognises a total of 130 species, based on the data collected in this survey and historical records. Amongst them, 93 native fish species were observed in this fish survey, including eight new records (See Table 1; note ‘+’). Thirty-four historically recorded species, unsampled in this field surveys, are contained in the updated checklist (Note ‘○’). Other 20 historically recorded species are excluded, including six being synonymised with other species (Note ‘S’), seven misidentified or having an erroneous record in the lake (Note ‘M’), four experiencing taxonomical changes (Note ‘T’) and three having unconfirmed records (Note ‘U’). A number of nomenclatural changes also occur for valid species included in the updated checklist (20 species). Taxonomic comments were appended to discuss its validity and occurrence where relevant.

#### Acipenseridae & Polyodontidae

The Acipenseridae has two representatives in the lake, namely *Acipensersinensis* Gray, 1835 and *A.dabryanus* Duméril, 1869, while the Polyodontidae is presented only by a single species *Psephurusgladius*. All three large-sized sturgeons were not collected in Lake Dongting during this field survey. One juvenile individual (4340 mm SL, 566.0 g) of *A.sinensis* was collected from East Dongting Lake during the 2012–2013 field survey (unpublished data). The specimen is likely a captive-bred individual released into the wild. This conservation measure has been implemented in the upper Chang-Jiang Basin for nearly twenty years (Du et al. 2013). Nichols (1928) was the first to report on the existence of *A.dabryanus* in Lake Dongting. Liang and Liu (1959, 1966) included the sturgeon in their species inventories of the lake. This species has vanished in Lake Dongting since the Gezhouba Dam was constructed across the Chang-Jiang mainstem (Zhang and Cao 2021a). Likely, *A.dabryanus* became highly depleted as no individuals have been collected in the river as from 1995 (Zhang et al. 2017). The capture record on *P.gladius* showed a similar trend to that of *A.dabryanus* as no records of Chinese paddlefish have been reported since 1995 (Zhang et al. 2020a).

#### Anguillidae

*Anguillajaponica* Temminck & Schlegel, 1846, a delicious food fish of economic importance in China and even across the Globe, is the only representative of the family in Lake Dongting. Historically, the lake and its affluents were utilised by this catadromous fish as feeding grounds (Anonymous 1980), but it is hardly seen in fish capture presently (Liu et al. 2013). One small individual (295 mm SL, 33.9 g) was captured at Chenglingji Channel during this field survey. It might be an individual which escaped from reservoirs where cage culture was used to farm this fish, in terms of local fishermen.

#### Clupeidae & Tetraodontidae

The family Clupeidae and Tetraodontidae are each represented in Lake Dongting by a single species. The two diadromous fishes, *Tenualosareevesii* and *Takifuguobscurus* (Abe, 1949), are hardly seen in this lake so far. The last capture of *T.reevesii* (one individual) was at Jiangsu provincial section of the Chang-Jiang in 1998 (Liu et al. 2002). *Takifuguobscurus* is occasionally encountered in the lower Chang-Jiang Basin so far (Wang et al. 2016; Chen et al. 2020).

#### Engraulidae

This family has only two representatives in Lake Dongting: *Coiliabrachygnathus* and *C.nasus* Temminck & Schlegel, 1846. So far, *C.brachygnathus* abounds in this lake where it is a delicious food fish of economic importance, but *C.nasus* is a rarely encountered fish. *Coilianasus* is even regarded to have been extinct due to anthropogenic interferences for nearly two decades (Wang et al. 2016); however, this anadromous fish has recently been found to persist in Lake Dongting (Xuan et al. 2020).

#### Salangidae

This family has four representatives in Lake Dongting: *Hemisalanxprognathus*, *Neosalanxbrevirostris* (Pellegrin, 1923), *N.jordani* Wakiya & Takahashi, 1937 and *Protosalanxhyalocranius* (Abbott, 1901). So far, the taxonomy of Chinese icefishes still remains controversial (Fu et al. 2005; Zhang et al. 2007). Based on the latest taxonomic advances of this family, three formerly recorded species were removed from the updated checklist. *Neosalanxtaihuensis* Chen, 1956 was treated as a synonym of *N.brevirostris* (Zhang et al. 2007). *Hemisalanxbrachyrostralis* (Fang, 1934) was synonymised with *H.prognathous* and so was *Neosalanxoligodontis* Chen, 1956 with *N.jordani* (Guo et al. 2011).

#### Catostomidae

This family has a single representative in China: *Myxocyprinusasiaticus* (Bleeker, 1864). *Myxocyprinusasiaticus* had long been considered as a migratory fish (Anonymous 1976). Nevertheless, Zhang and Zhao’s (2001) examination on collection specimens found that all individuals caught from the mid-lower Chang-Jiang Basin were small-sized, but large-sized individuals came from the upper reaches of this river, so concluding that *M.asiaticus* may be not a migratory species. One specimen (382 mm SL and 675.1 g) of this species was captured at the estuary of the Xiang-Jiang into Lake Dongting during our field survey. It is probably a captive-bred individual released into the Xiang-Jiang at Hengyang section yearly, according to local fishermen.

#### Cyprinidae

The Cyprinidae, as traditionally delimited, contains species with one to three rows of pharyngeal teeth, barbels present or absent and Weberian apparatus (Chen 1998; Nelson et al. 2016). A recent re-classification of the Cypriniformes was provided by Tan and Armbruster (2018), based on Yang et al.’s (2015a) phylogenetic relationships of this order inferred from both mitochondrial and nuclear genes. The Cyprinidae*s. l.* splits into ten families, namely Acheilognathidae, Cyprinidae*s. str.*, Danionidae, Gobionidae, Leptobarbidae, Leuciscidae, Sundadanionidae, Tanichthyidae, Tincidae and Xenocyprididae. The Cyprinidae*s. str.* is further subdivided into eleven subfamilies, i.e. Acrossocheilinae, Barbinae, Cyprininae, Labeoninae, Poropuntiinae, Probarbinae, Schizopygopsinae, Schizothoracinae, Smiliogastrinae, Spinibarbinae and Torinae. Amongst them, four subfamilies have their representatives in this lake: Acrossocheilinae, Cyprininae, Labeoninae and Spinibarbinae.

##### Acrossocheilinae

This subfamily was newly erected to include species currently designated to *Folifer*, *Onychostoma* and *Acrossocheilus* (Yang et al. 2015a; Tan and Armbruster 2018), but its generic classification still needs in-depth study. According to historical records (Tang and Qian 1979; Li 2006), *Onychostoma* has two representatives in Lake Dongting: *Onychostomasimum* (Sauvage & Dabry de Thiersant, 1874) and *O.rarum* (Lin, 1933). The two rheophilic fishes were not caught in this survey, though.

##### Cyprininae

Previously, *Cyprinuscarpio* Linnaeus, 1758 was extensively utilised as the available specific name for the common carp widespread in China. This species, however, is currently regarded as the endemic species of Europe (Kottelat and Freyhof 2007). The East Asian populations of the common carp represent a distinct species from *C.carpio*. The available specific name for it is *C.haematopterus* Temminck & Schlegel, 1846 (Zhou et al. 2003), a junior synonym of *C.rubrofuscus* Lacepède, 1803 (Kottelat 2006, 2013). Specimens previously reported by Li (2006) as *Procyprisrabaudi* (Tchang, 1930) from Lake Dongting are possibly misidentified. This species is a rheophilic fish usually found in headwaters of rivers, but not in the lentic environment (Zhang and Zhao 2016).

##### Labeoninae

This subfamily has four representatives in Lake Dongting: *Decorustungting*, *Cirrhinuscirrhosus* Bloch, 1795, *C.molitorella* (Valenciennes, 1844) and *Pseudogyrinocheilusprochilus* (Sauvage & Dabry de Thiersant, 1874). The first species were firstly designated to *Sinilabeo* Rendahl, 1933 (Wu 1977; Tang et al. 2001) and later moved into *Bangana* Hamilton, 1822 (Zhang and Chen 2006). Recently, Zheng et al. (2019) assigned this species, along with *Banganadecora* (Peters, 1881) from the Zhu-Jiang Basin, *B.lemassoni* (Pellegrin & Chevey, 1936) from the Red River Basin and *B.rendahli* (Kimura, 1934) from the upper Chang-Jiang Basin, to their own genus named as *Decorus*. Both *Cirrhinuscirrhosus* and *Pseudogyrinocheilusprochilus* are two new records of this lake. The former was introduced into China as cultured fish from India during the 1990s (Wang and Zhang 2021); it, like *C.molitorella* in southern China, has widely been farmed as food fish for cultured Mandarin fish (Ye et al. 2016). Individuals of two *Cirrhinus* fishes, caught from Lake Dongting in this field survey, probably escaped from farming waters. The latter *P.prochilus* was collected at Chenglingji, the outlet channel from Lake Dongting into the Chang-Jiang mainstem. This means that the species has an extended distribution in this lake. The rheophilic fish is mainly found in the upper Chang-Jiang and Zhu-Jiang presently (Zhang 1994; Zheng et al. 2010). It was even recorded from the Li-Shui, Yuan-Jiang and Xiang-Jiang (Liang and Liu 1966; Anonymous 1980; Cao et al. 2012).

##### Spinibarbinae

The subfamily is represented in Lake Dongting by a single species: *Spinibarbuscaldwelli* (Nichols, 1925). *Spinibarbuscaldwelli* was previously recognised as *S.hollandi* Oshima, 1919 (Chu and Chen 1989; Yue 2000), a species widespread in Asian mainland (Tang et al. 2005). Indeed, *S.hollandi* is endemic to Taiwan Island of China (Tang et al. 2005). The available scientific name for Asian mainland specimens of this species is *S.caldwelli* (Tang et al. 2005). *Spinibarbussinensis* Bleeker, 1871 is a species mainly found in the upper Chang-Jiang Basin (Chu and Chen 1989; Ding 1994; Yue 2000; Zhang and Zhao 2016). It was also reported from Lake Dongting by Tang and Qian (1979) and Li (2006). This identification, nevertheless, needs confirmation when specimens become available.

#### Xenocyprididae

The family is the dominant group of the ichthyofauna of Lake Dongting, with 30 species identified from 22 genera: *Aristichthys* Oshima, 1919 (one species), *Chanodichthys* Bleeker, 1860 (five), *Ctenopharyngodon* Steindachner, 1866 (one), *Culter* Basilewsky, 1855 (one), *Distoechodon* Peters, 1881 (one), *Elopichthys* Bleeker, 1860 (one), *Hemiculter* Bleeker, 1860 (two), *Hypophthalmichthys* Bleeker, 1860 (one), *Luciobrama* Bleeker, 1870 (one), *Megalobrama* Dybowski, 1872 (two), *Mylopharyngodon* Peters, 1881 (one), *Ochetobius* Günther, 1868 (one), *Opsariichthys* Bleeker, 1863 (one), *Parabramis* Bleeker, 1864 (one), *Plagiognathops* Berg, 1907 (one), *Pseudobrama* Bleeker, 1870 (one), *Pseudolaubuca* Bleeker, 1864 (two), *Sinibrama* Wu, 1939 (one),*Squaliobarbus* Günther, 1868 (one), *Toxabramis* Günther, 1873 (one), *Xenocypris* Günther, 1868 (two) and *Zacco* Jordan & Evermann, 1902 (one). The large majority of these species are widespread in the lowlands of south or east China.

Several previously-recorded species from Lake Dongting have synonymisations or taxonomic changes. *Xenocyprisargentea* Günther, 1868 was synonymised with *X.macrolepis* Bleeker, 1871 (Kottelat 2013). *Xenocyprismicrolepis* Bleeker, 1871 had been referred to *Plagiognathops* Berg, 1907 (Kottelat 2013). *Zaccoacanthogenys* (Boulenger, 1901) had long been synonymised with *Zaccoplatypus* (Temminck & Schlegel, 1846) until Wang (2019) and Zhu et al. (2020) revalidated it. The type locality of *Z.platypus* is in Japan (Liu et al. 2011), but *Z.acanthogenys* occurs in the mid-lower Chang-Jiang Basin. Specimens under the name of *Sinibramawui* (Rendahl, 1933) from Lake Dongting are referred to as *S.macrops* (Günther, 1868), following Xie et al. (2003) and Zhang et al. (2004). Specimens, previously recognised as *Megalobramaskolkovii* Dybowski, 1872, from the lake are identified as *M.mantschuricus* (Basilewsky, 1855), following Vasil’eva and Makeeva (2003) and Bogutskaya et al. (2008).

The taxonomy of three genera *Chanodichthys* Bleeker, 1860, *Culter* Basilewsky, 1855 and *Cultrichthys* Smith, 1938 is hitherto in a chaotic status in Chinese literature. The type species of *Chanodichthys* is *Leptocephalusmongolicus* Basilewsky, 1855 [type locality: China: Mongolia (presently Inner Mongolia Province) and Manchuria (now northeast China)], that of *Culter* is *C.alburnus* Basilewsky, 1855 [type locality: China: rivers flowing into the Gulf of Tschili (today’s Hebei Province)] and that of *Cultrichthys* is *C.brevicauda* Günther, 1868 (type locality: Taiwan, China). Bănărescu (1997) synonymised *Cultrichthys* with *Culter*. This synonymisation was subsequently accepted by some researchers (Bogutskaya and Naseka 2004; Bogutskaya et al. 2008). The type species of *Culter*, though, was misplaced in *Cultrichthys* in Chinese literature (Luo 1994; Luo and Yue 1996; Chen 1998). This misplacement can be traced back to Yi and Zhu (1959), who took it for granted that *Cultrichthyserythropterus* (Basilewsky, 1855), as indicated by the species name, is the available scientific name for the species with pink pectoral, pelvic and anal fins. This character, along with a long keel extending along the mid-line of the chest and belly, is typical for *Culteralburnus* (Bogutskaya et al. 2008). *Cultrichthyserythropterus* (*sensu*Chen 1998) is, thus, the misidentification of *Culteralburnus* (*sensu*Chen 1998) and vice versa. *Culter*, as here delimited, includes two species: *C.alburnus* and *C.compressocorpus* Yih & Chu, 1959. All other species currently placed to *Culter* by Chinese authors should be referred to as *Chanodichthys*.

#### Gobionidae

Twenty-five species of gudgeons from Lake Dongting are placed in 12 genera, namely *Abbottina* Jordan & Fowler, 1903 (one species), *Coreius* Jordan & Starks, 1905 (one), *Gobiobotia* Kreyenberg, 1911 (four), *Hemibarbus* Bleeker, 1860 (two), *Microphysogobio* Mori, 1934 (one), *Paracanthobrama* Bleeker, 1864 (one), *Pseudogobio* Bleeker, 1860 (one), *Pseudorasbora* Bleeker, 1860 (one), *Rhinogobio* Bleeker, 1870 (three), *Sarcocheilichthys* Bleeker, 1860 (three), *Saurogobio* Bleeker, 1870 (six) and *Squalidus* Dybowski, 1872 (one). Most of these species are often seen in the mid-lower Chang-Jiang basin or even lowland areas of the southern China.

*Sarcocheilichthys* is represented in Lake Dongting by three species, namely *S.nigripinnis* (Günther, 1873), *S.tungtingensis* Nichols & Pope, 1927 and *S.sinensis* Bleeker, 1871. Our ongoing taxonomy of this genus demonstrates that *S.kiangsiensis* Nichols, 1930 occurs only in the lake Poyang system and that specimens, formerly identified as this species from Lake Dongting, belong to *S.tungtingtensis* (An 2020). A critical revision of *Sarcocheilichthys* from China is underway; the species diversity of this genus has been highly underestimated so far. *Saurogobio* is presently represented in Lake Dongting by six species, namely *S.dabryi* Bleeker, 1871, *S.dumerili* Bleeker, 1871, *S.gracilicaudatus* Yao & Yang, 1977, *S.gymnocheilus* Lo, Yao & Chen, 1998, *S.lissilabris* Bănărescu & Nalbant, 1973 and *S.xiangjiangensis* Tang, 1980. Both *S.gracilicaudatus* and *S.xiangjiangensis* are new records and so is *P.vaillanti* (Sauvage, 1878). The species status of *S.lissilabris* was suspected by some researchers (Wu 1977; Chen 1998) or even it was synonymised with *S.gymnocheilus* (Dai et al. 2014). Tang et al. (2018) considered it as a valid species on the basis of molecular evidence and their examination on its type. Two historically documented species are removed from the updated species checklist: *Coreiusguichenoti* (Sauvage & Dabry de Thiersant, 1874) and *Squalidusnitens* (Günther, 1873). The former is hitherto found only in the upper Chang-Jiang (Zhang et al. 2019; Liu et al. 2020a) and the latter, whose type locality is in Shanghai City (Günther 1873), has not been found in Lake Dongting for decades.

Four species of *Gobiobotia* were formerly reported from Lake Dongting: *G.filifer* (Garman, 1912), *G.meridionalis* Chen & Cao, 1977, *G.nicholsi* Bănărescu & Nalbant, 1966 and *G.pappenheimi* Kreyenberg, 1911. The first species is to date endemic to the Chang-Jiang Basin downstream of Yibin City. The second species had long been treated as a subspecies of *G.longibarba* Fang & Wang, 1931 until Chen (1998) regarded it as a full species. It is extensively known from the middle reaches of the Chang-Jiang and Zhu-Jiang Basins (Chen 1998; Tang et al. 2001; Zhang and Zhao 2016). The two species were collected from this lake during this field survey. The third species was originally described from Lake Dongting (Bănărescu and Nalbant 1966), but later synonymised with *G.filifer* (Wu 1977). Our ongoing taxonomy of Chinese species of *Gobiobotia* shows that *G.nicholsi* is a valid species of the lake Dongting system, but it is so far known merely by its type specimens. Although Bănărescu and Nalbant (1966) reported on the occurrence of *G.pappenheimi* and *Xenophysogobioboulengeri* (Tchang, 1929) in Lake Dongting, no additional specimens have since been collected. Generally, *G.pappenheimi* (type locality: northern China: Tianjin City) is mainly found in the Hai-He and Huang-He (Wang 1984) and *X.boulengeri* (type locality: southwest China: Sichuan Province) occurred in the upper Chang-Jiang Basin (Zhang et al. 2019). Our photograph examination indicated that specimens, identified by Bănărescu and Nalbant (1966) as *G.pappenheimi* and *X.boulengeri* from Lake Dongting, are conspecific with *G.nicholsi*. Nevertheless, this identification still needs confirmation when topotypical specimens become available. Provisionally, these two species are here regarded to have an erroneous record in the Lake. Recently, a new species from Lake Dongting is here found, based on morphological and molecular evidence (Chen et al. 2022b). Therefore, the eight-barbel gudgeons have four representatives in the lake: *Gobiobotiafilifer*, *G.lii*, *G.meridionalis* and *G.nicholsi*.

#### Acheilognathidae

The bitterlings have eleven representatives in Lake Dongting: *A.macropterus* (Bleeker, 1871), *A.barbatulus* Günther, 1873, *A.macromandibularis* Doi, Arai & Liu, 1999, *A.polylepis* (Wu, 1964), *A.gracilis*, *A.barbatus* Nichols, 1926, *A.chankaensis* (Dybowski, 1872), *A.tonkinensis* (Vaillant, 1892), *A.hypselonotus* (Bleeker, 1871), *Rhodeusocellatus* (Kner, 1866) and *R.sinensis* Günther, 1868. The first one was formerly misidentified as *A.taenianalis* (Günther, 1873) (Tang and Qian 1979; Li 2006); however, *A.taenianalis* has been shown to be a junior synonym of *A.macropterus* (Kottelat 2013). The second and third bitterlings are new records for this lake (Doi et al. 1999; Li 2013). *Acheilognathusimberbis* Günther, 1868, previously documented from Lake Dongting (Ru 2008), is removed from the species checklist. Its type locality remains unclear, but it is reportedly present in the lower Chang-Jiang Basin so far (Li et al. 2016c; Zhang et al. 2020b). Moreover, this species has not been found in the Lake Dongting system over the past several decades. Although Yu et al. (2005) reported on its distribution in Xiang-Jiang, the identification still needs confirmation.

#### Botiidae

This family is so far represented in Lake Dongting by five species, three of which are from *Leptobotia* [*L.citrauratea* (Nichols, 1925), *L.rubrilabris* (Dabry de Thiersant, 1872) and *L.taeniops* (Sauvage, 1878)] and two from *Parabotia* [*P.fasciata* Dabry de Thiersant, 1872 and *P.banarescui* (Nalbant, 1965)]. Nichols (1925b) reported on the occurrence of *Botiarubrilabris* in the Lake Dongting system and described *B.purpurea* and *B.citrauratea* as two new species from the lake. The three sympatrically existing congeneric species were later transferred to *Leptobotia* where *B.citrauratea* and *B.purpurea* were synonymised, respectively with *L.elongata* and *L.taeniops* (Bleeker, 1870) (Chen 1980; Kottelat 2004, 2012). Recently, *L.citrauratea* was resurrected from the synonym of *L.elongata*, based on examination of the type and morphological data (Bohlen and Šlechtová 2017). Guo and Zhang (2021) also affirmed that *L.citrauratea* survives in Lake Dongting (type locality). Only a single small-sized individual of *L.rubrilabris* was collected by Anonymous (1980) in this lake. Our field survey yielded no specimens of this species. Likely, it has been extirpated in Lake Dongting. The taxonomy of *Leptobotia* species from China needs a critical revision.

#### Bagridae

The taxonomy of the bagrid catfishes from China is notoriously poorly understood. This family is represented in Lake Dongting by two genera: *Hemibagrus* Bleeker, 1862 and *Tachysurus* Lacepède, 1803. Species previously referred to *Mystus* Scopoli, 1777 are misidentification of *Hemibagrus* in Chinese literature (Liu et al. 2013; Yuan et al. 2019; Yang et al. 2020). All species, formerly placed in *Pelteobagrus* Bleeker, 1864 and *Pseudobagrus* Bleeker, 1858, are currently referred to *Tachysurus* (Ng and Freyhof 2007; Kottelat 2013) and so are Chinese species formerly placed in *Leiocassis* Bleeker, 1857 (Cheng and Zhang 2012), which is in fact a genus endemic to Southeast Asia (Ng and Kottelat 2007).

The Bagridae is represented in Lake Dongting by 10 species, namely *Tachysuruscrassilabris* (Günther, 1864), *T.dumerili* (Bleeker, 1864), *T.eupogon* (Boulenger, 1892), *T.mica* (Gromov, 1970), *T.nitidus* (Sauvage & Dabry de Thiersant, 1874), *T.sinensis* Lacepède, 1803, *T.ussuriensis* (Dybowski, 1872), *T.vachellii* (Richardson, 1846), *T.zhangfei* Shao, Cheng & Zhang, 2021 and *Hemibagrusmacropterus* Bleeker, 1870. *Tachysurusdumerili* is a senior subjective synonym of *T.longirostris* Günther, 1864 (Kottelat 2013). Specimens of *T.mica* were formerly misidentified as the juveniles of other catfishes owing to their small size (Chu et al. 1999), but our ongoing taxonomy of Chinese *Tachysurus* indicates that it is a valid species. *Tachysurussinensis* is a senior subjective synonym of *T.fulvidraco* (Richardson, 1846) (Ng and Kottelat 2007). Specimens, previously recognised as *T.albomarginatus* (Rendahl, 1928), from Lake Dongting represent an undescribed species, which was named as *T.zhangfei* (Shao et al. 2021). Possibly, Ru’s (2012) specimens, under the name of *T.tenuis* (Günther, 1873), from Lake Dongting were misidentified as it is hitherto known merely from the type locality, Chongming Island, Shanghai City (Kottelat 2013; Cheng et al. 2021). This species is tentatively excluded from this updated species checklist.

#### Ictaluridae & Centrarchidae

The family Ictaluridae and Centrarchidae are each represented in Lake Dongting by a single species. *Ictaluruspunctatus* (Rafinesque, 1818) and *Micropterussalmoides* (Lacepède, 1802), introduced as cultured fishes to China, are sporadically found in the lakes from southern China (Li et al. 2016d).

#### Sinipercidae

This family is so far represented in Lake Dongting by four species of the genus *Siniperca* Gill, 1862: *S.chuatsi* (Basilewsky, 1855), *S.knerii* Garman, 1912, *S.roulei* Wu, 1930 and *S.scherzeri* Steindachner, 1892 (Tang and Qian 1979; Li 2006; Li 2013). This third perch was previously assigned to *Coreosiniperca* Fang & Chong, 1932, but this genus has been shown to be invalid (Liu and Chen 1994).

#### Gobiidae

Five gobies of *Mugilogobius* Smitt, 1900 and *Rhinogobius* Gill, 1859 were previously recorded from Lake Dongting: *M.myxodermus* (Herre, 1935), *R.brunneus* (Temminck & Schlegel, 1845), *R.cliffordpopei*, *R.giurinus* Gill, 1859 and *R.similis* Gill, 1859 (Tang and Qian 1979; Li 2006). Specimens, under the name of *R.giurinus*, have been shown to be misidentification of *R.similis* (Suzuki et al. 2016; Suzuki et al. 2017). The current identification of *R.brunneus* from this lake remains suspicious. Its type locality is in Japan (Temminck and Schlegel 1845). Chinese specimens of this goby were referred to as different species (Wu and Zhong 2008). Nevertheless, whether the species exists in Chinese freshwaters remains unsolved yet. Temporarily, the goby is removed from this updated checklist. Thus, only three gobies are here recognised from Lake Dongting: *M.myxodermus*, *R.cliffordpopei* and *R.similis*.

## ﻿Discussion

### ﻿Species diversity

Lake Dongting, as the second-largest river-connected freshwater lake lying within the floodplain areas of the mid-lower Chang-Jiang Basin, supports diversified freshwater fish species. A total of 130 fish species is here reported from the Lake. This number accounts for ca. 31.48% of the total freshwater fishes of the Chang-Jiang Basin where 413 native species were recently documented (Zhang and Cao 2021a). According to the recently-published book entitled “The fish fauna of Hunan Province”, the Dongting Lake system harbours up to 218 freshwater fish species (Wu et al. 2021). The lake alone contributes to 59.63% of the total number of freshwater fishes from the system. In addition to serving as favourable habitats of the Yangtze finless porpoise (Huang et al. 2017) and the crucial stopover and breeding grounds of plentiful migrating birds (Fang et al. 2006; Zou et al. 2019), this lake is also used as sanctuaries or nursery grounds by numerous larvae of drifting-egg-spawning or potamodromous fishes like four major Chinese carps, as spawning grounds by some anadromous fishes like *Coilianasus* and *Tenualosareevesii* and as feeding grounds by some catadromous fishes like *Anguillajaponica* and *Takifuguobscurus* (Dou and Jiang 2000). Evidently, Lake Dongting is the key biodiversity area of this lake system or the Chang-Jiang Basin.

The total number of freshwater fish species of Lake Dongting given in this updated checklist is actually comparable to that of Lake Poyang, the first-largest river-connected floodplain lake of the mid-lower Chang-Jiang Basin, where a total of 136 fish species has been recorded so far (Zhang and Li 2007; Yang et al. 2015b; Fang et al. 2016). This number seems to be higher than that of Lake Dongting, but remains doubtful. From the latest species checklist of freshwater fishes from the Gan-Jiang—the largest river flowing into Lake Poyang, 36 historically recorded species were removed (Wang and Zhang 2021). Amongst them, at least ten species were contained in checklists of fish species of Lake Poyang by Zhang and Li (2007) and Fang et al. (2016); these ten species were also components of the ichthyofauna of Lake Poyang system compiled by Huang et al. (2013) and Hu et al. (2019). Both Lakes Dongting and Poyang support rich fish species diversity that is unmatched by any other lake in the mid-lower Chang-Jiang Basin, such as Lake Chao (54 fish species, Guo et al. 2007), Lake Tai (107, Zhu et al. 2007), Lake Hongze (88, Lin et al. 2013) or Lake Hong (84, unpublished data) and far higher than that of lakes located in the Yunnan-Guizhou Plateau (Yuan et al. 2010). This can be plausibly explained by uniqueness of these two large-sized floodplain subtropical lakes: the permanent lateral hydrological connection with the Chang-Jiang mainstem and co-existence of lentic and lotic environments. The assembly mechanism maintaining fish community within Lake Dongting has been addressed in Chen et al. (2022a).

The present study shows that fish species diversity of Lake Dongting remains insufficiently understood. The number of species, collected from the Lake in this survey, is lower compared with the frontrunners (Liang and Liu 1959, 1966; Tang and Qian 1979; Li 2006). Eight newly-recorded native species are added likely due to the multiple sampling methods used and three seasonal samplings during our survey from 2017 to 2019. More sampling efforts lead to the discovery of higher species richness (Hughes et al. 2021; Pompeu et al. 2021). Twenty historically-recorded species are excluded from the checklist, mainly due to the following reasons: (1) Species misidentification. This is the case for seven species which do not exist in the lake presently, namely *Coreiusguichenoti*, *Gobiobotiapappenheimi*, *Leptobotiaelongata*, *Procyprisrabaudi*, *Pseudobagrustenuis* (= *Tachysurustenuis*), *Spinibarbussinensis* and *Xenophysogobioboulengeri*; (2) Taxonomic alteration. Species, formerly identified as *Cyprinuscarpio*, *Sarcocheilichthyskiangsiensis*, *Spinibarbushollandi* and *Zaccoplatypus* from this lake or China, are now referred to as *Cyprinusrubrofuscus*, *Sarcocheilichthystungtingtensis*, *Spinibarbuscaldwelli* and *Zaccoacanthogenys*, respectively; (3) Unconfirmed records. Whether *Acheilognathusimberbis*, *Rhinogobiusbrunneus* and *Squalidusnitens* occur in Lake Dongting remains controversial; (4) Synonymisation. The following six species are to date regarded as invalid: *Acheilognathustaenianalis*, *Hemisalanxbrachyrostralis*, *Neosalanxoligodontis*, *Neosalanxtaihuensis*, *Rhinogobiusgiurinus*, and *Sinibramawui*. It is apparent that problems with the current identification of some fish species in Lake Dongting still remains.

This checklist includes 20 species which experienced nomenclatural alterations, viz. *Banganatungting* (= *Decorustungting*), *Coreosinipercaroulei* (= *Sinipercaroulei*), *Culteralburnus* (= *Chanodichthyserythropterus*), *Culterdabryi* (= *Chanodichthysdabryi*), *Cultermongolicus* (= *Chanodichthysmongolicus*), *Culteroxycephaloides* (= *Chanodichthysoxycephaloides*), *Culteroxycephalus* (= *Chanodichthysoxycephalus*), *Cultrichthyserythropterus* (= *Culteralburnus*), *Gobiobotialongibarbameridionalis* (= *Gobiobotiameridionalis*), *Leiocassisargentivittatus* (= *Tachysurusmica*), *Leiocassiscrassilabris* (= *Tachysuruscrassilabris*), *Leiocassislongirostris* (= *Tachysurusdumerili*), *Mystusmacropterus* (= *Hemibagrusmacropterus*) *Pelteobagruseupogon* (= *Tachysuruseupogon*), *Pelteobagrusfulvidraco* (= *Tachysurussinensis*), *Pelteobagrusnitidus* (= *Tachysurusnitidus*), *Pelteobagrusvachellii* (= *Tachysurusvachellii*), *Pseudobagrusalbomarginatus* (= *Tachysuruszhangfei*), *Pseudobagrusussuriensis* (= *Tachysurusussuriensis*), *Xenocyprismicrolepis* (= *Plagiognathopsmicrolepis*). Two species, *Gobiobotianicholsi* and *Sarcocheilichthystungtingensis* are, for the time being, regarded as valid. Their taxonomic status needs to be confirmed when specimens from their type locality (today’s East Dongting Lake) become available.

### ﻿Biodiversity conservation

Amongst 130 freshwater fish species of Lake Dongting, 12 (9.23% of the total) are labelled as threatened species in Zhang and Cao’s (2021a) assessment of the Red List of Chinese freshwater fishes, viz. *Acipensersinensis* (CR), *A.dabryanus* (CR), *Anguillajaponica* (EN), *Decorustungting* (EN), *Leptobotiarubrilabris* (VU), *Luciobramamacrocephalus* (CR), *Myxocyprinusasiaticus* (CR), *Ochetobiuselongatus* (CR), *Onychostomararum* (VU), *Psephurusgladius* (CR), *Rhinogobioventralis* (EN) and *Tenualosareevesii* (CR) (see Table 3). Three species, *Psephurusgladius*, *Acipensersinensis* and *A.dabryanus*, are also listed in the Appendices II of the Convention on International Trade in Endangered Species (CITES 2019). *Psephurusgladius* was declared to have been functionally extinct in the Chang-Jiang Basin, due to a permanent lack of reproduction or recruitment since 1993 (Zhang et al. 2020a). No wild individuals on *A.dabryanus* have been monitored since 1995 (Zhang et al. 2017). The critically endangered status of *A.sinensis* was mainly owing to a dramatic decline in population after 2000 (Zhang and Cao 2021a). Field surveys conducted from 2002 to 2009 found a trend of a drastic decrease in its juvenile population year by year (Wang et al. 2011; Wu et al. 2015). No spawning individuals were monitored from 2013 to 2015 into the Chang-Jiang mainstem downstream of the Gezhouba Dam (Wu et al. 2017; Zhang et al. 2020a), therefore indicating that the population of this freshwater megafauna species is extremely impacted by river damming (Zhang et al. 2017). Only one small individual of *A.sinensis* was collected from Lake Dongting during 2012 (unpublished data). This clearly means that the lake can be utilised as nursery or feeding grounds by the juveniles and, hence, plays an important role in the conservation of the sturgeon. Nevertheless, the young sturgeon is also likely the captive-bred juveniles released into the upper Chang-Jiang, given that restocking, one salvaging measure taken to conserve this fish, has been in place for several decades (Du et al. 2013). Two imperilled species, *Anguillajaponica* (EN) and *Myxocyprinusasiaticus* (CR) were collected in this field survey, indicating that both still persist here. More attention should be paid to the remaining threatened fish species unsampled in this survey. Whether they eluded capture or have been extirpated, their populations are in a continuous decline and salvaging actions should be adopted immediately.

**Table 3.** Endemics of the mid-lower Chang-Jiang Basin and protected fish species in Lake Dongting.

Species	CITES	China	Hunan	IUCN	Endemics
*Psephurusgladius*	√	I		CR	
*Acipensersinensis*	√	I		CR	
*Acipenserdabryanus*	√	I		CR	
*Coilianasus*			√	LC	
*Coiliabrachygnathus*				DD	√
*Tenualosareevesii*		I	√	CR	
*Neosalanxbrevirostris*			√	DD	
*Anguillajaponica*				EN	
*Myxocyprinusasiaticus*		II	√	CR	
*Onychostomasimum*			√	NT	
*Onychostomararum*			√	VU	
*Spinibarbuscaldwelli*			√	LC	
*Luciobramamacrocephalus*		II	√	CR	
*Ochetobiuselongatus*			√	CR	
*Decorustungting*			√	EN	√
*Megalobramaamblycephala*				LC	√
*Acheilognathusmacropterus*				LC	√
*Acheilognathushypselonotus*				LC	√
*Acheilognathusmacromandibularis*				LC	√
*Microphysogobiotungtingensis*			√	DD	√
*Saurogobiogracilicaudatus*				LC	√
*Saurogobioxiangjiangensis*			√	LC	
*Rhinogobioventralis*		II		EN	
*Leptobotiacitrauratea*				DD	√
*Leptobotiarubrilabris*		II		VU	
*Sinipercaroulei*			√	NT	
*Channaasiatica*			√	LC	
*Macropodusopercularis*			√	NT	
Total	3	8	15		9

Five species are also on the latest List of Key Protected Wild Animals in China, namely *Leptobotiarubrilabris*, *Luciobramamacrocephalus*, *Myxocyprinusasiaticus*, *Rhinogobioventralis* and *Tenualosareevesii* (Anonymous 2021). *Tenualosareevesii* is of importance for capture fisheries, particularly in the mid-lower Chang-Jiang Basin. The population of this anadromous fish had been in remarkable decrease as from 1992 when Wan’an Dam was constructed across the Gan-Jiang, where its spawning grounds were located (Tang et al. 1993; Liu 2002). During the past twenty years, no individuals have been collected (Wang and Zhang 2021). The fish, like *Psephurusgladius*, has probably been extinct in the Chang-Jiang Basin (Zhang and Cao 2021a). *Myxocyprinusasiaticus* is rarely encountered in Lake Dongting due to a sharp decline in population resulting from anthropogenic disturbances (Fang et al. 2006). One small individual of 382 mm SL, which was caught during our field survey, is likely a captive-bred juvenile released into the wild to restock its population. *Luciobramamacrocephalus* used to be widely distributed in southern China, but this food fish of high value has become an occasionally-encountered species. The carnivorous fish has long been regarded as the target species to be eradicated as its juveniles prey on fries of other farmed fishes, thus having negative impacts on lake or reservoir fisheries. Deliberate removal of this apex predator was mainly responsible for its current endangerment status. *Rhinogobioventralis* was initially described from Lake Dongting (Sauvage and Thiersant 1874), but the gudgeon has vanished since Liang and Liu’s (1959, 1966) report on its existence in the lake. *Leptobotiarubrilabris*, originally described from the upper Chang-Jiang Basin, was also recorded from Lake Dongting (Nichols 1925b). The latest report on its survival in the lake was Anonymous (1980), who caught a single specimen of 80 mm SL. Field survey of fishes conducted from 2014 to 2019 into Lake Dongting yielded no specimens of this fish (Guo and Zhang 2021). Likely, it has already been extirpated in this system.

Besides three species (*Luciobramamacrocephalus*, *Myxocyprinusasiaticus* and *Tenualosareevesii*), there are another 12 species currently included in Hunan provincial key protected wildlife list (The Forest Department of Hunan Province 2015): *Channaasiatica*, *Coilianasus*, *Decorustungting*, *Macropodusopercularis*, *Microphysogobiotungtingensis*, *Neosalanxbrevirostris*, *Ochetobiuselongatus*, *Onychostomararum*, *O.simum*, *Saurogobioxiangjiangensis*, *Sinipercaroulei* and *Spinibarbuscaldwelli* (Table 3). No specific conservation actions, however, have been in place for these species. It is worth pointing out that, except for the *Channaasiatica* and *Macropodusopercularis*, all these species seem to be of local economic importance in the mid-lower Chang-Jiang Basin or Lake Dongting system.

Lake Dongting harbours nine fish species endemic to the mid-lower Chang-Jiang Basin, namely *Acheilognathushypselonotus*, *A.macromandibularis*, *A.macropterus*, *Coiliabrachygnathus*, *Decorustungting*, *Leptobotiacitrauratea*, *Megalobramaamblycephala*, *Microphysogobiotungtingensis* and *Saurogobiogracilicaudatus*. These species have a high risk of being imperilled by anthropogenic perturbation. More efforts should be dedicated to monitor their population size and trend. *Decorustungting*, a popular food fish of local economic importance in Lake Dongting system before 1980s, is currently restricted only to some sections of the Yuan-Jiang and Zi-Shui, two affluents of Lake Dongting (Bian et al. 2011). Owing to a sharp decrease in population over the past 30 years, this rheophilic species was assessed as Endangered (EN) in the latest assessment of Chinese freshwater fish Red List (Zhang and Cao 2021a). No doubt, salvaging actions should be taken to conserve this species. All these species, except *D.tungting*, were not included in this Red List. Nevertheless, two fishes were listed as Data Deficient (DD): *Coiliabrachygnathus* and *Microphysogobiotungtingensis*. *Leptobotiacitrauratea* can also be assessed in this category and was recently revalidated (Guo and Zhang 2021). These three species are possibly under the same threat as *D.tungting* and, thus, deserve special attention.

Thirty-four historically documented fish species were not collected from Lake Dongting during this field survey; their fate is of particular concern. These species fall within five categories. The first one is migrating species, like *Acipenserdabryanus*, *A.sinensis*, *Coilianasus*, *Psephurusgladius*, *Tenualosareevesii* and *Takifuguobscurus*. The main reasons for the extirpation of the first four species in the lake are mentioned above. Although the last two species eluded capture during this field survey, both were reportedly collected in exceptional years (Ren et al. 2015; Wang et al. 2016; Chen et al. 2020). Since 1970s, more and more dams have been built across the affluents of Lake Dongting and also the Chang-Jiang mainstem (Wang et al. 2019a). The blockage of migration ways and the shrinkage of favourable habitats were the key factors leading to a sharp decrease in the population of the two diadromous fishes (Wang et al. 2016). Small population size makes it difficult for them to migrate for such a long distance from the estuary of Chang-Jiang into Lake Dongting, particularly when all fishes, being of economic importance in the river, were under high pressure from fishing during the past 20 years. The second category is such potamodromous or drifting-egg-spawning fishes as *Luciobramamacrocephalus*, *Ochetobiuselongatus*, *Pseudolaubucaengraulis*, *Rhinogobiocylindricus*, *R.ventralis* and *Saurogobiodumerili*, which are susceptible to dam construction. River damming makes inundated reaches shift from lotic to lentic habitat, which have adverse impacts on the spawning of these species. The third category is rheophilic species such as *Decorustungting*, *Leptobotiarubrilabris*, *Lepturichthysfimbriatus*, *Onychostomararum*, *O.simum*, *Spinibarbuscaldwelli*, *Tachysurusussuriensis* and *Zaccoacanthogenys*. Their extirpation in Lake Dongting is mainly attributed to river damming in its affluents, which not only led to a remarkable decline in the population of these fishes, but also blocked their short migration into the lake. The fourth category is bitterlings, such as *Acheilognathusbarbatus*, *A.chankaensis*, *A.hypselonotus* and *A.tonkinensis*, which depend on freshwater mussels for spawning. The absence of these bitterlings in Lake Dongting may be related to the decrease or disappearance of mussels caused by degrading water quality or sand extraction (Meng et al. 2018; Liu et al. 2020b; Wang and Zhang 2021). The fifth category includes some fishes of economic value, such as *Distoechodontumirostris* Peters, 1881, *Neosalanxbrevirostris*, *N.jordani*, *Plagiognathopsmicrolepis* and *Protosalanxhyalocranius*. These fishes eluded capture mainly due to small population size led by overfishing and habitat loss or degradation. Overall, most of the unsampled fish species during this field survey have ecologically specialised preferences, for example, migratory, rheophilic, carnivorous, drifting-egg-producing or mussel-dependent. These fishes are susceptible to human disturbances and, thus, can act as biological indicators of aquatic ecosystem health. Their lack of samples clearly indicates that the freshwater ecosystem of Lake Dongting has been severely threatened by human perturbations including river damming, overfishing, habitat degradation and sanding dredging.

The Chang-Jiang basin is an area with over 400 million residents, highly impacted by anthropogenic interferences. It is also the most rapidly growing area of China’s economic development. The loss of aquatic diversity and, thus, its ecological service function in this river is becoming a pressing challenge. It is urgently needed to take practical actions to conserve the freshwater ecosystem of the Chang-Jiang Basin. To this end, the Chinese government made a decision of implementing the conservation measure of ‘ten-year fishing ban’ in all natural water bodies of the mainstem and major tributaries of the Chang-Jiang since 2020 (Pan and Liu 2021). Whether it is an effective protection action for conserving the fish diversity of Lake Dongting is of much public concern. In this context, adequate information about the current status of fish diversity, including species composition, distribution, population size and imperilled status, is an urgent requirement in the future to answer the question. This updated species checklist will be very useful for further biodiversity analysis and conservation of freshwater fishes from Chang-Jiang.

## Supplementary materials

Supplementary material 1 Table S1 Data type Table (docx. file) Explanation note Table S1. Geographical coordinates of 20 sampling sites in Lake Dongting. https://binary.pensoft.net/file/704839This dataset is made available under the Open Database License (http://opendatacommons.org/licenses/odbl/1.0/). The Open Database License (ODbL) is a license agreement intended to allow users to freely share, modify, and use this Dataset while maintaining this same freedom for others, provided that the original source and author(s) are credited.Xiao Chen
