# Supplementary material for: ﻿Updated species checklist of fishes from Lake Dongting in Hunan Province, South China: Species diversity and conservation
Source: Zookeys. 2022 Jun 23;1108:51–88. doi: 10.3897/zookeys.1108.79960 (PMC9848865; doi:10.3897/zookeys.1108.79960)
Supplement: Supplementary material 1 — Table S1 [file zookeys-1108-051_article-79960__-s001.docx]

**Table S1** Geographical coordinates of 20 sampling sites in Lake Dongting

| **Sites** | **Name** | **Latitude** | **Longitude** | **Sub-lake** | **Environment** |
| --- | --- | --- | --- | --- | --- |
| 1 | Chenglingji | 29°26′09.73″N | 113°08′43.87″E | East | Lotic |
| 2 | Junshan | 29°27′50.46″N | 113°00′02.74″E | East | Lotic |
| 3 | Liumenzha | 29°27′36.33″N | 112°45′35.26″E | East | Lentic |
| 4 | Nanyuepo | 29°22′36.09″N | 113°05′21.59″E | East | Lotic |
| 5 | Luohanzhou | 29°18′28.76″N | 113°02′40.07″E | East | Lotic |
| 6 | Lujiao | 29°09′29.56″N | 113°00′05.03″E | East | Lotic |
| 7 | Leishixiang | 28°59′03.03″N | 112°57′57.17″E | East | Lotic |
| 8 | Henglinghu | 28°49′14.29″N | 112°47′17.58″E | South | Lentic |
| 9 | Wugongmiao | 28°48′07.89″N | 112°52′50.80″E | South | Lotic |
| 10 | Lulintan | 28°48′04.88″N | 112°53′28.42″E | South | Lotic |
| 11 | Zhangshugang | 28°33′40.99″N | 112°49′02.63″E | South | Lotic |
| 12 | Liaodaokou | 28°51′39.02″N | 112°33′37.87″E | South | Lentic |
| 13 | Wanzihu | 28°51′15.65″N | 112°31′48.61″E | South | Lentic |
| 14 | Zijiangkou | 28°50′23.92″N | 112°21′16.58″E | South | Lotic |
| 15 | Dongnanhu | 28°52′30.37″N | 112°22′53.68″E | South | Lentic |
| 16 | Xiaohezui | 28°50′52.84″N | 112°19′08.92″E | South | Lentic |
| 17 | Kongjiaohu | 28°48′54.99″N | 112°17′20.48″E | West | Lentic |
| 18 | Jiangjiazui | 29°00′05.24″N | 111°58′31.08″E | West | Lentic |
| 19 | Guantouzhen | 28°50′51.76″N | 112°11′49.05″E | West | Lotic |
| 20 | Maocaojie | 29°04′01.77″N | 112°19′12.60″E | West | Lotic |
